# Characterizing the Content and Structure of AAV Capsids by Size Exclusion Chromatography and Orbitrap-Based Charge Detection-Mass Spectrometry

**DOI:** 10.1021/jasms.5c00074

**Published:** 2025-06-26

**Authors:** Kanchan Pathak, Gustavo Perrotti, Stephen J. Rosa, Graham Robinett, Lance Kasper, Qiangwei Xia, Carlos R. Escalante, Fabio P. Gomes

**Affiliations:** † 6889 Virginia Commonwealth University, Department of Chemistry, Richmond, Virginia 23284, United States; ‡ 6426 Agilent Technologies, Santa Clara, California 95051, United States; § CMP Scientific Corp, Brooklyn, New York 11226, United States; ∥ 6889 Virginia Commonwealth University, School of Medicine, Department of Physiology and Biophysics, Richmond, Virginia 23298, United States

**Keywords:** adeno-associated virus (AAV), gene therapy, recombinant AAV (rAAV), native size exclusion chromatography (nSEC), orbitrap-based charge detection-mass spectrometry (CD-MS)

## Abstract

Adeno-associated virus (AAV) is currently the most widely used vector in gene therapy applications. However, a significant challenge in the manufacturing process of recombinant AAV (rAAV) is the presence of empty capsids, oligomers, aggregates, and partially filled capsids. These components do not provide any therapeutic benefit but add to the overall viral load, which could increase immunogenicity and reduce transduction efficiency. Here, we present a strategy that utilizes size exclusion chromatography (SEC) equipped with multiangle light scattering (MALS) and a diode-array detector (DAD), followed by orbitrap-based charge detection-mass spectrometry (CD-MS). The SEC step was used to separate AAV capsids (non-aggregates) from oligomers, aggregates, and low molecular weight contaminants. In the second step, we employed direct CD-MS infusion using capillary electrophoresis with a sheath liquid (MS) interface. This approach facilitated automated, reproducible, sensitive, and robust CD-MS determination of empty-filled capsids, capsid oligomers, and encapsidated genomes. Importantly, the empty-to-filled capsid ratio was inaccurate without the SEC step. Together, our analytical platform offers a reliable and comprehensive approach for assessing the rAAV purity and characterizing key quality attributes, including capsid aggregation, capsid oligomerization, and genome packaging.

## Graphical abstract

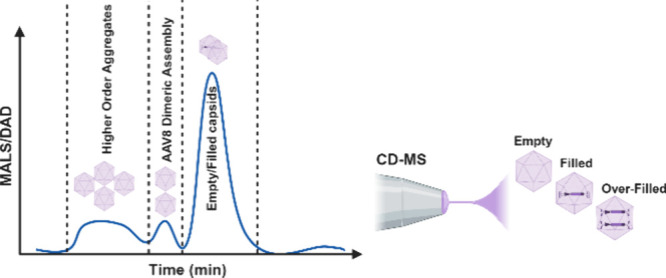

## Introduction

The recent success of AAV-based gene therapy applications has created a need for effective analytical tools with high separation efficiency and sensitivity for quality control during rAAV production.^1^ Currently, there are seven FDA/EMA-approved biologics using AAV technology to treat a diverse number of diseases and many more in Phase II/III clinical trials.^2,3^ AAV serotype 8 (AAV8) exemplifies the potential of rAAV biologics, demonstrating robust gene transduction and delivery to the liver in preclinical models, including primates and rodents. This serotype has gained considerable attention due to its relatively low immunogenicity. Numerous AAV8-based therapies are currently in clinical trials to deliver genes for hemoglobinopathies and other clinical disorders.^4–6^ Furthermore, studies have shown that AAV8 offers superior transduction and expression in the liver than other serotypes (e.g., AAV2).^4^

The AAV virus consists of a single-stranded DNA genome of ∼4.7 kb enclosed by an icosahedral capsid shell comprising three viral proteins, VP1, VP2, and VP3, at an approximately 1:1:10 ratio.^6^ A significant challenge in AAV gene therapy is the high vector doses needed to produce a therapeutic effect, partly due to the presence of empty capsids.^7–10^ Therefore, it is crucial to minimize empty capsids and other contaminants, as well as to implement a reliable and reproducible method for their detection. Doing so will enhance rAAV production and maximize its efficacy. In addition, AAV particles can aggregate under thermal stress or at high concentrations during manufacturing, storage, and use. For example, it is problematic to administer small volumes of concentrated rAAV vectors to certain sites (e.g., central nervous system) because increasing the concentration of AAV products can lead to aggregation. Furthermore, rAAV aggregation could also occur due to capsid oligomerization. Thus, a detailed understanding of AAV capsid oligomerization is critically important for the development of AAV-based gene therapies. Indeed, rAAV aggregation is a significant concern. The aggregation of AAV particles can lead to inconsistencies in AAV content, losses during purification, increased immunogenicity, and therapeutic inefficacy.^10^ Importantly, AAV packages a single-stranded (ss) DNA genome, which is flanked by two inverted terminal repeats (ITRs). They are crucial for genome replication and packaging. Studies have shown that AAV capsids can be packaged with truncated genomes or unresolved inverted terminal.^11^ There is an unmet need for a fast and robust approach to guarantee the integrity of the packaged genome of interest (GOI).^12^ The AAV capsid content is one of the most critical quality attributes. rAAV-based therapies are dosed according to the VG titer. Indeed, determining the content of rAAV-based products represents a significant analytical challenge due to their aggregation propensity, high molecular weights, heterogeneity, limited sample amounts, and structural complexity.^10,13^

Common approaches to solve these limitations and to measure multiple AAV attributes include size exclusion chromatography (SEC)^14^ or ion-exchange chromatography (IEX)^1,15–17^ coupled to light scattering detectors^18,19^ or mass spectrometers.^20^ Although SEC is an attractive chromatographic strategy for monitoring aggregation, this technique is limited by its separation mechanism, which relies on differences in size in solution to achieve separation. IEX delivers high-resolution separations, but high-salt concentrations might affect the integrity of AAV assemblies.^21^ Furthermore, this new generation of biotherapeutics is much larger and more complex than previous antibody-based products. Native mass spectrometry (native MS) has been used for fast AAV analysis,^22,23^ but limitations associated with unresolved peaks in the *m*/*z* spectrum preclude the assignment of charge states.^24^ On the other hand, charge detection-mass spectrometry (CD-MS) enables sensitive analysis of heterogeneous AAV assemblies.^12,16,24–28^ This single-particle technique allows the determination of each ion’s mass by measuring (simultaneously) the mass–charge ratio and charge of individual ions.^24^ Thus, empty and filled ratios can be accurately quantified. Mass photometry (MP) has been used for the analysis of AAV capsids.^29,30^ This single-molecule technique offers high resolution, speed, and sensitivity.

Recently, Ebberink et al. compared MP and CD-MS techniques for their ability to characterize AAV capsids.^30^ In this study, both techniques were considered valuable tools for characterizing AAV capsids. While MP was highlighted as a simpler and faster technique, CD-MS was deemed to provide superior mass accuracy. Interestingly, the Marty and Jarrold groups have demonstrated the online coupling between SEC and CD-MS for the analysis of large proteins and virus-like particles.^31,32^ An automated CD-MS microfluidic platform has been reported for the analysis of megadalton biotherapeutics.^33^ In particular, automated workflows are needed for fast and reproducible assessment of key AAV quality attributes (e.g., capsid aggregation, genome packing, molecular weight, and content ratio). AAV-based products’ success relies on the AAV vectors’ homogeneity and purity. For quality assurance and safety purposes, it is critically important to evaluate the content of AAV products by separating AAV (nonaggregates) from its aggregates, oligomers, and other impurities.

Protein-based drugs can aggregate during development, manufacturing, or storage, leading to their toxicity and efficacy reduction.^34^ This critical quality attribute must be monitored. Offline native size exclusion chromatography (nSEC) has recently been used to fractionate small-sized protein assemblies from breast cancer cells.^35^ Although SEC is well-suited for separating and quantifying large biomolecules, it is unable to separate empty and full capsids because they have the same size in solution.^36,37^

Here, we demonstrate that offline nSEC in conjunction with multiangle light scattering (MALS) and diode-array detector (DAD) is an attractive option to identify and distinguish AAV (nonaggregates) from its aggregates, oligomers, and low molecular weight contaminants prior to orbitrap-based CD-MS analysis. The choice of suitable pore and particle sizes enabled baseline separation between AAV8 (nonaggregates and its aggregates, oligomers, and low molecular weight contaminants). nSEC fractions (AAV8 capsid monomers and AAV8 capsid dimers) were collected and infused at low nano flow-rate using capillary-electrophoresis (CE) with sheath liquid MS interface and ionized by nano electrospray ionization (ESI). CD-MS was used to measure individual capsid ions, determine the ratio of empty and filled AAV8-capsids, confirm the presence of oligomers, and define the molecular size of the GOI. Importantly, the nSEC step ensured an accurate measurement of the empty-to-filled capsid ratio. AAV8 capsid was selected as a testbed due to its promising clinical relevance.

## Methods

### Native Size Exclusion Chromatography (nSEC) Conditions

Ammonium acetate solution 7.5 M (A2706) was purchased from Sigma-Aldrich. AAV8 empty (AAV8-Empty [2 × 10^13^ vg/mL]) and full (AAV8-CMV-GFP [2 × 10^13^ vg/mL]) capsids were purchased from VIROVEK. The theoretical mass of the empty capsid was expected to be ∼3.7 MDa while the theoretical mass of the filled capsid was expected to contain one copy of single-stranded DNA (ssDNA, ∼2.4 kb or 803 kDa). AAV8 empty and full capsids were mixed (1:1, v/v). The AAV8 mixture was injected (6 replicates) into a 1260 Infinity II Bioinert LC system (Agilent Technologies). The mobile phase was 200 mM ammonium acetate. The AAV8 mixture was fractionated on an AdvanceBio SEC column (300 × 7.8 mm; 2.7 μm; 500 Å [Agilent Technologies]) using isocratic elution at a flow rate of 1.0 mL/min. Fractions (F2 and F3) were collected from ∼6–9 min at ∼0.5 min intervals. A volume of 50uL was injected into the chromatographic system. The elution profile was monitored by UV absorbances at 280 and 260 nm and the Agilent 1260 MALS at 20 different angles. The WinGPC software (Agilent Technologies) was used to process the data. The nSEC fractions were collected using a 1260 Bio FC-AS fraction collector (Agilent Technologies). The temperature of the fraction collector was set to 4 °C. Each fraction was then concentrated using a 100 kDa molecular weight cutoff filter for subsequent orbitrap-based CD-MS analysis. The concentration procedure was performed 1 time (12,000 × *g* for 5 min at 4 °C). UV absorbance measurements at 260 and 280 nm were used to determine the ratios of empty and full capsids (monomeric and dimeric capsid assemblies). The percentages of dimeric capsid assemblies in the AAV sample mixture were calculated using the formulas below. The peak area values were obtained from peaks in the chromatograms. Each peak area value used for calculation was obtained by averaging the peak areas from the six replicates for each chromatographic peak (*n* = 6).

$$Capsid\;monomer\;A_{260}/A_{280}\;ratio=\frac{Peak\;Area\left(Absorbance\;at\;260\;nm\right)}{Peak\;Area\left(Absorbance\;at\;280\;nm\right)}$$

$$Capsid\;\dim er\;A_{260}/A_{280}\;ratio=\frac{Peak\;Area\left(Absorbance\;at\;260\;nm\right)}{Peak\;Area\left(Absorbance\;at\;280\;nm\right)}$$

$$\%\;AAV8\;capsid\;\dim er\left(Absorbance\;at\;260\;nm\right)=\frac{Peak\;Area\left(\dim er\right)}{Peak\;Area\left(\dim er\right)+Peak\;Area\left(monomer\right)}\times 100$$

$$\%\;AAV8\;capsid\;\dim er\left(Absorbance\;at\;280\;nm\right)=\frac{Peak\;Area\left(\dim er\right)}{Peak\;Area(\dim er)+Peak\;Area\left(monomer\right)}\times 100$$

### Capillary Electrophoresis (CE)-Sheath Liquid Infusion

Samples were infused into the mass spectrometer using a CE system (ECE001, CMP Scientific Corp) at a constant flow rate (∼80 nL/min [100 mbar]) on a PS2 neutral coating capillary (∼100 cm × 50 μm i.d., CMP Scientific Corp). The CE device was interfaced with the mass spectrometer using an EMASS-II CE-MS ion source (CMP Scientific Corp). A borosilicate glass emitter (CMP Scientific Corp) was used as nano ESI spray emitter, and the spray voltage ranged from 2.2 and 2.5 kV. The temperature of the autosampler was set to 4 °C. The background electrolyte (BGE) was composed of 200 mM ammonium acetate while the sheath buffer was composed of 25 mM ammonium acetate. Samples were infused (triplicate measurements) for about 10 min (non-aggregates) and 30 min (oligomers). Fraction 3 (F3) from nSEC (AAV8 capsid monomers) was also infused using different pressures (50 mbar [∼40 nL/min], 100 mbar [∼80 nL/min], 200 mbar [∼160 nL/min], 300 mbar [∼240 nL/min], and 500 mbar [∼400 nL/min]). The flow rate was experimentally estimated (triplicates) by measuring the time necessary for the BGE to fill the entire capillary at a constant pressure. We collected the BGE into a vial for 10 min, then we calculated the flow rate by dividing the collected volume by the time collected. Each sample (without nSEC fractionation) was desalted using a 100 kDa molecular weight cutoff filter for subsequent orbitrap-based CD-MS analysis. The desalting procedure was performed 5 times (12,000 × *g* for 5 min at 4 °C) to minimize salt effects. To evaluate the effect of nSEC purification, we quantified both the F3 and whole sample mixture (no nSEC fractionation) using a BCA assay kit and diluted them with 200 mM ammonium acetate to approximately 43 μg/μL for subsequent CD-MS analysis.

### Orbitrap-Based Charge Detection-Mass Spectrometry (CD-MS) Conditions

CD-MS measurements were performed on an Orbitrap Q Exactive with Ultra-High Mass Range and Direct Mass Technology (QE-UHMR-DMT) mass spectrometer (Thermo Fisher Scientific). Data was collected in DMT mode. Sulfur hexafluoride (SF_6_) was used as a collision gas. The mass spectrometer was operated in positive mode. The inlet capillary temperature was set to 275 °C. Trapping gas pressure was set to 2. The extended trapping was set to 10. The in-source trapping was set to −10 V and −120 V. The injection time was set to 200 ms (capsid monomers) and 500 ms (capsid oligomers). The resolution was set to 200,000 (capsid monomers) and 25,000 (capsid oligomers). Detector *m*/*z* was set to high.

### Data Analysis

Raw files were processed using STORIboard (version 1.0.24204.1 [Proteinaceous]). A calibration curve in “high mass” was used to assign charge states. Carbonic anhydrase, alcohol dehydrogenase, pyruvate kinase, and β-galactosidase were used to construct the calibration curve. Processing templates were used to interpret the data and calculate the AAV percentage. After processing the data, the resolution was adjusted between 0.001 and 0.012. The percentage of each AAV capsid assembly in the nSEC fractions and samples was obtained by averaging them in each of the three replicates using the software STORIboard.

### Statistical Analysis

Results are shown as mean ± confidence value. The percentage of AAV capsid assemblies in the nSEC fractions and samples was determined by averaging it in the three replicates. The mean percentage and confidence value of each species among the three (CD-MS) or six (nSEC) replicates were calculated.

## Results and Discussion

To ensure a thorough characterization of the purity of rAAV particles, we have meticulously designed a comprehensive two-pronged protocol. This protocol serves two primary purposes: first, it identifies and characterizes a range of contaminants that may affect the quality of the rAAV preparations, and second, it analyzes the empty-to-full capsid ratio. By evaluating these critical factors, we aim to provide an in-depth assessment of particle integrity, which is essential for the success of any downstream applications.

### Native Size-Exclusion Chromatography (nSEC) of AAV8

To test our protocol, we obtained empty and filled AAV8 capsids from a commercial source, allowing us to control the mixing ratios. First, we prepared full and empty AAV8 capsids at a 1:1 ratio (v/v) and analyzed this mixture using offline nSEC prior to CD-MS analysis. To enhance the efficiency of the nSEC step, the chromatographic column was judiciously selected. Virus-like particles possess various surface characteristics, including charged and hydrophobic regions, which can lead to nonspecific interactions with the stationary phase and often result in peak tailing. To mitigate these issues, we opted for a column with a hydrophilic coating to prevent such undesirable interactions. Additionally, a column with a small particle size (2.7 μm) was required to increase separation efficiency. Given that AAV has a compact structure and measures approximately 20–26 nm (200–260 Å) in size, we selected a column with a pore size of 500 Å. Ultimately, the selected chromatographic column has all the required features to enable a baseline separation of single AAV capsids from their aggregates, oligomers, and lower molecular weight species.

Importantly, the flow path of the chromatographic system can also be a source of undesired secondary interactions.^36^ We addressed this issue using a bioinert instrument with a metal free flow path. Our instrument, equipped with both a 20-angle MALS and DAD detectors, provided complementary information on AAV8 assemblies. MALS demonstrated superior sensitivity for detecting AAV8 capsid dimers and higher-order aggregates compared to UV absorbance (Figure 1A), consistent with its inherent sensitivity to macromolecular size and concentration. The peak eluting between ∼6 and 7.5 min (Fraction 2 [F2], Figure 1) corresponds to AAV8 capsid dimers. It exhibited enhanced MALS signal. The DAD facilitated the detection of low molecular weight species, which might correspond to truncations of VP proteins or ssDNA (Figure 1A).^12,38^

**Figure 1:**
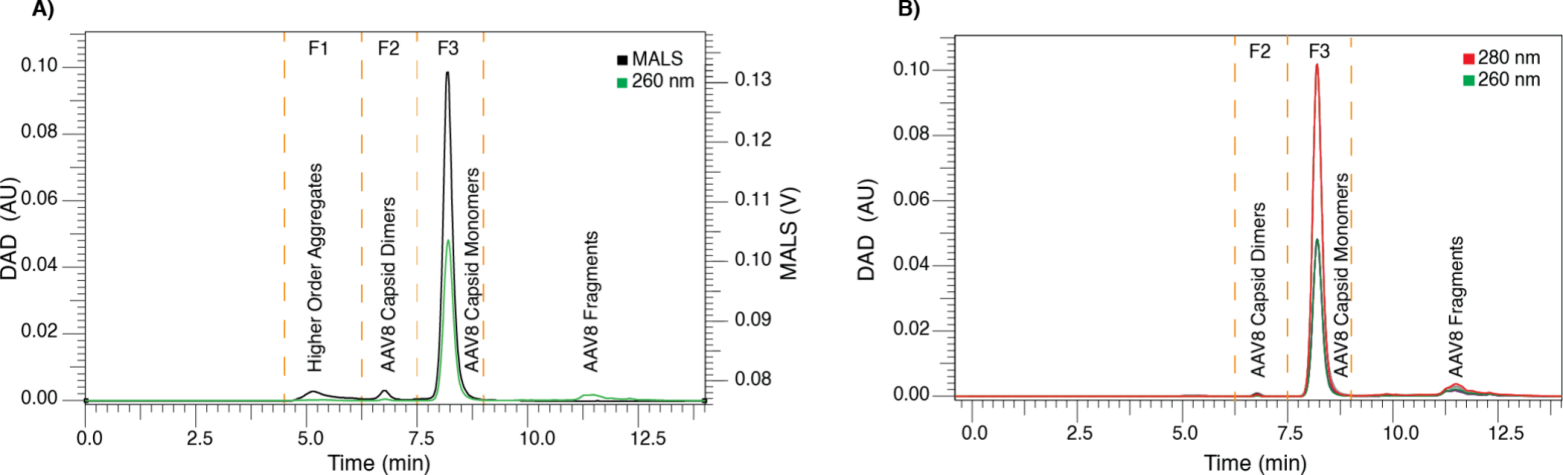
**nSEC chromatographic profiles (MALS and DAD) of the AAV8 mixture (empty:filled)**. (A) AAV8 sample mixture (6 overlapped replicates) detected with MALS (black) and DAD at 260 nm (green). (B) AAV8 sample mixture (6 overlapped replicates) detected with DAD at 260 nm (green) and DAD at 280 nm (red).

### F* – nSEC Fraction

The relative proportion of AAV8 dimers, calculated as a percentage of total AAV signal (see Methods and Table 1), was quantified using peak area integration.

**Table 1:** Evaluation of the AAV8 Mixture (Empty:Filled Ratio), AAV8 Empty Capsids, and AAV8 Filled Capsids^a^

Sample	E (*x̅* ± CV)	F (*x̅* ± CV)	E:F (*x̅* ± CV)	OF (*x̅* ± CV)
AAV8 capsid monomers (E:F – nSEC/CD-MS)^b^	54 ± 1	46 ± 1	----	----
AAV8 capsid monomers (E:F – CD-MS)^b^	63 ± 4	37 ± 4	----	----
AAV8 capsid monomers (E – CD-MS)	98 ± 1	----	----	----
AAV8 capsid monomers (F – CD-MS)	5 ± 3	94 ± 4	----	----
AAV8 capsid monomers (E:F – nSEC/CD-MS)	42 ± 2	39 ± 2	----	19 ± 1
AAV8 capsid dimers (E:F nSEC [260 nm])	----	----	0.9 ± 0.01	----
AAV8 capsid dimers (E:F nSEC [280 mn])	----	----	1 ± 0.01	----
AAV8 capsid dimers (E:F nSEC/CD-MS)	1 ± 0.3	3 ± 1	----	----

a *x̅*, mean (%) ± CV, confident value; F, full; E, empty; OF, overfilled.

b The same concentration.

Importantly, the DAD enables AAV detection at multiple wavelengths (280 and 260 nm, Figure 1B). Notably, the DAD’s capability for multiwavelength detection (280 and 260 nm, Figure 1B) allowed us to assess the A260/A280 peak area ratio for both AAV8 capsid monomers and dimers. Leveraging the principle that nucleic acids exhibit maximal absorbance at 260 nm and proteins at 280 nm,^39^ we used this ratio as an indicator of relative ssDNA to protein content. A260/280 values below 0.5 suggest a higher proportion of empty capsids.^40^ For the capsid monomers (F3), prepared as a 50/50 mixture of full and empty capsids, the A260/A280 ratio was measured at 0.5. The capsid dimers (F2) exhibited at 0.4. These results indicate that both monomeric and dimeric fractions have higher proportions of empty than full capsids. Peak area ratios were calculated as mean and confidence value (*n* = 6). As illustrated in Figure 1, the overall consistency of AAV sample mixture across 6 replicates reflected the reproducibility and robustness of our nSEC strategy. It is important to note that using the 260/280 ratio for quantitative analysis requires careful calibration of well characterized standards relevant to the AAV product being measured. An intrinsic limitation of the method is that coeluting partial filled or overfilled AAVs are not accounted for. Thus, to validate our analytical workflow, we determined the AAV8 content in the sample mixture using orbitrap-based CD-MS. Our chromatographic system is also equipped with a bioinert fraction collector, which enabled us to isolate specific components of interest (AAV8 capsid monomers [F3] and AAV8 capsid dimers [F2]) for further CD-MS analysis.

### Orbitrap-Based Charge Detection-Mass Spectrometry (CD-MS) of AAV8

CD-MS is uniquely positioned to interrogate heterogeneous macromolecules where charge state distribution and isotopic resolution cannot be achieved using conventional native MS. Furthermore, this single molecule technique has been successfully used to study higher-order aggregates.^34,41^ Our nSEC step used a mobile phase that is compatible with CD-MS. Therefore, the fractions were ready for direct nano ESI infusion. The nSEC fractions (Figure 1A) were infused using CE, ionized via nano ESI, and charge-detected. This workflow allowed us to measure individual ions and obtain molecular mass of AAV8 oligomers, encapsidated genomes, empty capsids, and capsids with different amounts of genomic material and conformations. CE with a sheathless MS interface has shown proficiency for infusion of intact proteins.^42^ However, it is often difficult to maintain a stable spray using this interface due to its poor electrical contact at the capillary tip.^43^ In this workflow, CE with a sheath liquid MS interface was used as an infusion platform, as it delivers optimal spray stability with a fully controlled and stable flow rate. We evaluated the influence of the pressure or flow rate on the reproducibility and sensitivity of our CD-MS approach. As described in the Method section, the F3-nSEC was infused into the mass spectrometer at different flow rates (triplicates). It is widely accepted that reduced flow rates can enhance both ionization and transmission efficiencies in nano ESI, and thus, increase the ion signal intensity. As illustrated in Figure 2, enhanced ion signal (empty and filled capsids) was observed when the infusion flow rate was decreased. This phenomenon is attributed to reduced suppression effects and improved signal-to-noise ratios. Surprisingly, the overall ion signal (overfilled capsids) remained roughly constant across these triplicate measurements (Figure 2). The most likely explanation is that all available overfilled capsid ions were effectively transmitted. Thus, varying the flow rate did not significantly impact the overfilled ion signal, as the droplet formation and subsequent ion release may have already reached a plateau. The overall consistency of the spray stability (at all different flow rates) across triplicate measurements reflected the reliability, reproducibility, robustness, and sensitivity of our CD-MS approach.

**Figure 2:**
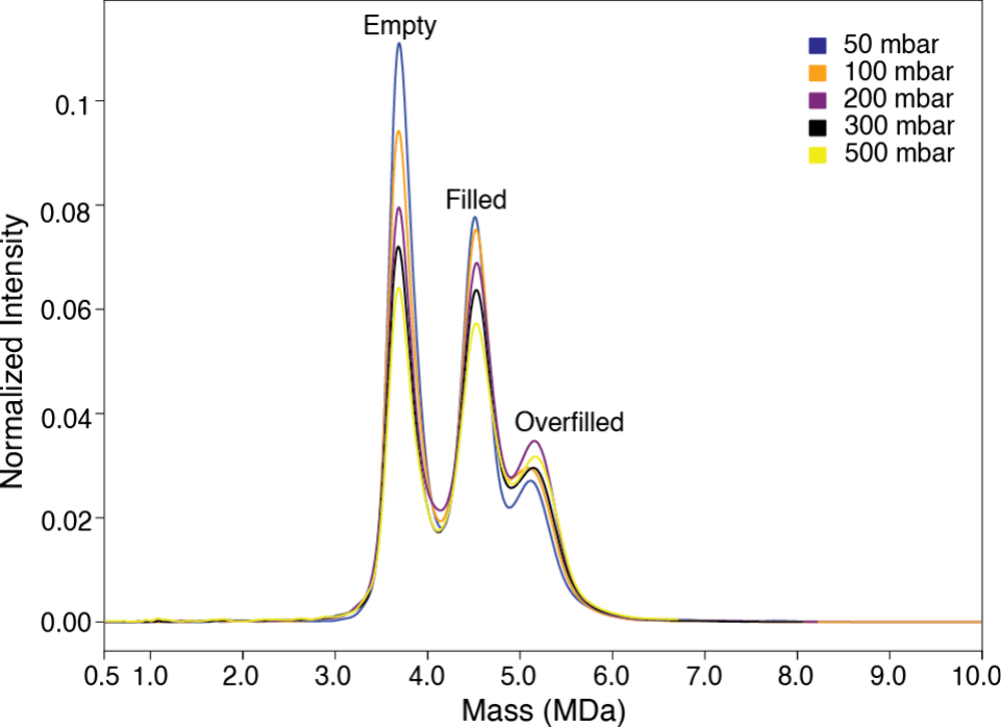
**Signal intensity of the F3-nSEC sample at different pressures/flow rates (** ***n*** **= 3).**

Further experiments were conducted at a pressure of 100 mbar, which corresponds to ∼80 nL/min. Importantly, a controlled nanoflow rate can maximize the ionization efficiency in nano ESI by focusing the droplet emission zone toward the MS inlet.^42^ Automated CE infusions were performed using a capillary with a large inner diameter, which enabled direct ionization of AAV8 capsids without clogging problems. Clogging is a common issue in native MS and CD-MS experiments. As the infusion flow rate was fully controlled by our CE platform, the MS signal was stable, ensuring the robustness and reproducibility of our CD-MS strategy. Due to the extended acquisition time required by this MS-based method, long spray stability is required in CD-MS experiments. Importantly, sample consumption in CE is negligible, allowing us to introduce samples into the mass spectrometer for extended durations continually. Importantly, a neutral-coated capillary was used for infusions to avoid retention of these large virus-like capsids in the capillary wall.^44^ Sulfur hexafluoride (SF_6_) was selected as a collision gas in the mass spectrometer. Heavy and polyatomic gases such as SF_6_ have shown proficiency at improving collisional cooling, focusing, and transmission of high *m*/*z* ions.^45^ rAAV preparations include AAV aggregates (dimers or other higher-order species), empty particles, and filled particles. In addition, if the ssDNA genome is smaller than 4.5 kbs, rAAV particles with more than one genome can be identified.^46^ Figure 3 shows the CD-MS spectrum of the F3 sample, which corresponds to the AAV8 capsid monomers. The raw spectrum of the Figure 3 is available in the Supporting Information (Supporting Figure 1). The peak at 3.7 MDa represents an empty capsid (Figure 3A). Since the purchased AAV8 was packaged with a genome of approximately 2.5 kbs (half of the full-sized AAV genome), some particles were packaged with two copies of the genome (truncated). The peak at 4.5 MDa is consistent with a capsid filled with one copy of single-stranded DNA (ssDNA, ∼803 kDa, Figure 3A). The peak at 5.1 MDa is consistent with a capsid that is packaged up to the packing capacity or a truncated double-stranded (ds) DNA or two copies of ssDNA, including a truncated ssDNA (Figure 3A). Here, we use the term “overfilled” to describe a capsid that is packaged with two copies of the 2.5 kbs ssDNA (truncated) or truncated dsDNA. The percentages of empty, filled, and overfilled capsids are shown in Table 1. The observed mass of the overfilled capsid was approximately 200 kDa lighter than the theoretical mass of an overfilled capsid with two copies of ssDNA (truncated) or dsDNA. A reasonable explanation for this mass shift is an ssDNA truncation. This DNA’s degradation event has been previously reported.^11,12^ As illustrated in Figure 3B, the charge versus mass scatter plot of the empty, filled, and overfilled capsids has very similar charges, indicating that the genetic material is packed inside the capsid. The cluster of ions at ∼3.7 MDa (∼150 charges) represents the empty capsid. While the cluster of ions at ∼4.5 MDa (∼150 charges) is attributed to the filled capsid, the cluster of ions at ∼5.1 MDa (∼150 charges) represents the overfilled capsid. As shown in Figure 3B, there are two charge populations. One is centered at ∼150, the other is centered at ∼50. These elementary charges are likely due to capsid monomers with different conformations, with the lower-charged cluster indicating more compact structures.

**Figure 3:**
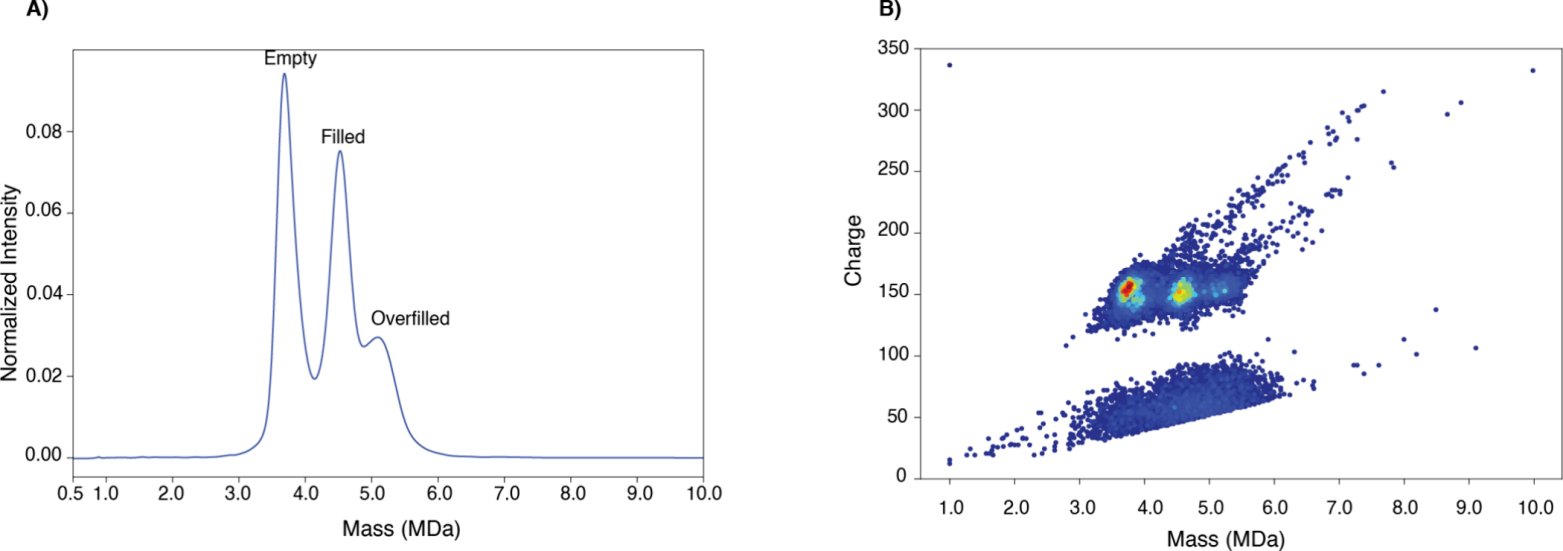
**CD-MS analysis of the AAV8 mixture (empty:filled) after nSEC fractionation.** (A) CD-MS spectrum of the F3-nSEC (AAV8 capsid monomers, *n* = 3). (B) Charge versus mass scatter plot of the F3-nSEC (AAV8 capsid monomers, *n* = 3).

In addition to intact capsid masses, our CD-MS approach confirmed the masses of encapsidated genome by ejecting them from both filled and overfilled particles. By using controlled in-source trapping (IST, −120 V, Figure 4, red spectrum), we ejected the genomes from both filled and overfilled capsids. In addition to the empty capsid and a small portion of the filled capsid, peaks at ∼770 and 819 kDa were observed. We attribute each peak to two different ssDNA structures (Figure 4). As mentioned previously, the theoretical mass of the GOI is about 803 kDa. The masses of the measured peaks are slightly smaller (33 Da) or larger (16 Da) than the sequence mass. While the increased mass shifts could be attributed to DNA methylations or adduct ions, the decreased mass shift could be related to ssDNA truncation.^12,47^ Also, structural DNA instability during orbital motion in the analyzer section may affect the mass accuracy.^47^ A peak at ∼1.2 MDa, with almost twice the mass of the GOI, was assigned as a truncated version of dsDNA. As mentioned previously, AAV8 capsids can encapsulate genomes up to 4.7 kb. As illustrated in Figure 4 (blue spectrum), the masses of these genomes were not observed when IST was set to −10 V. In addition, filled and overfilled capsids were observed, confirming the ejection of the genomes from the capsids. Importantly, the ejection of the encapsidated genome with controlled IST does not require a sample pretreatment, and thus, it offers a fast and effective strategy to measure of truly encapsidated genomes.

**Figure 4:**
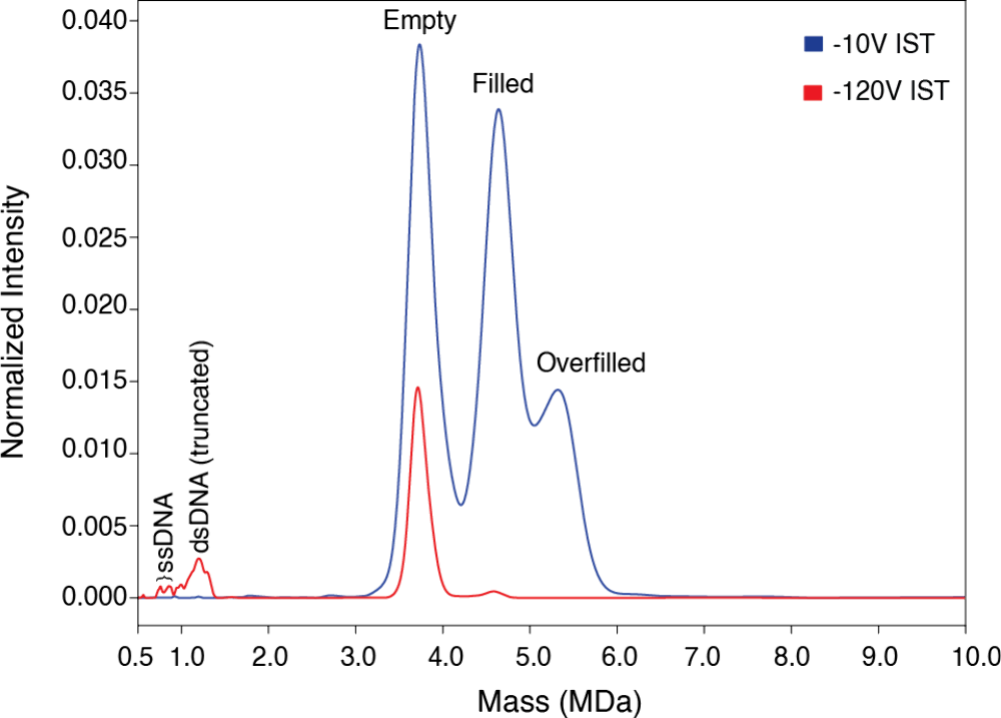
Analyses of the encapsidated genome by CD-MS.

As control samples, AAV8 empty and filled capsids were individually analyzed (at the same concentration) by CD-MS without nSEC fractionations (Figure 5A,B). The raw spectra of Figure 5A,B are available in the Supporting Information (Supporting Figure 2). The filled particle was found to carry empty particles and particles with truncated dsDNA. Table 1 illustrates the percentages of empty, filled, and overfilled in each capsid.

**Figure 5:**
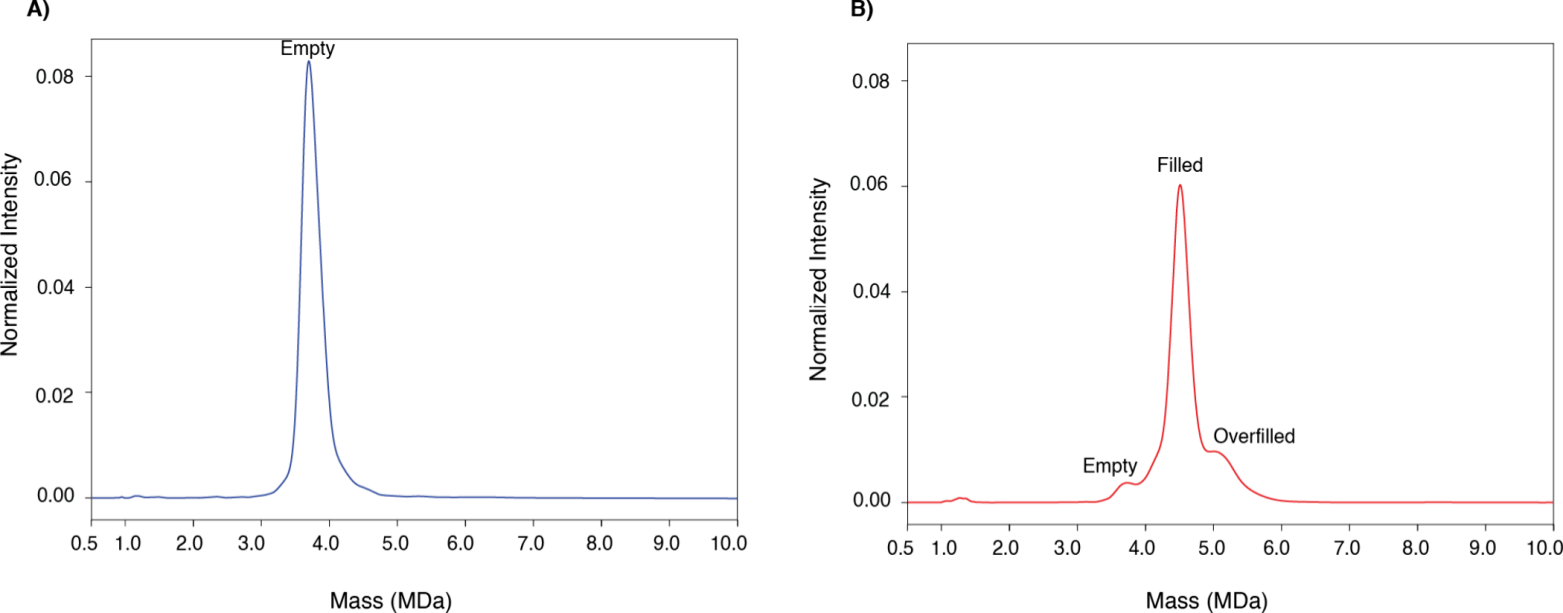
**CD-MS analysis of the AAV8 empty capsid and AAV8 filled capsid without nSEC fractionation.** (A) CD-MS spectrum of the empty capsid. (B) CD-MS spectrum of the filled capsid.

To confirm the presence of capsid dimers, we analyzed the sample F2 (Figure 1A) by CD-MS. As mentioned previously, we injected the whole sample mixture into the chromatographic system 6 consecutive times. As shown in Figure 1A, the signal intensity and separation window of the nSEC method were found to be highly reproducible, allowing for enrichment of capsid dimers. Obtaining efficient ionization at ultralow-flow rates was particularly important to this CD-MS workflow, not only to improve ionization efficiency and reduce ion suppression effects but also to detect low-abundant species such as AAV capsid oligomers. As illustrated in Figure 6, our CD-MS approach detected the AAV8 capsid dimers, including empty and filled capsids. The two charge clusters (centered at 300) at around 7.4 and 9.0 MDa (Figure 6A) represent the dimers of empty and filled capsids, respectively. It is important to note that the charges (centered at 300) of the capsid dimers are similar, which indicates that the genome material is packed inside the capsid dimer. Monomeric capsids with different conformations were also observed. The dimers also seem to have a lower charge population centered at ∼120 charges, which could be related to dimeric capsids with more compact structures. A small cluster of charges (centered at ∼450 charges) at ∼11 MDa could be associated with the presence of capsid trimmers. Figure 6B illustrates the charge versus mass/charge (*m*/*z*) distribution plot of the dimer capsids. Figure 6C shows the mass distribution of the capsid dimers that were measured by CD-MS. The peak at ∼7.4 MDa represents an empty dimeric capsid. The peaks at ∼8.7 and ∼9.6 MDa lack conclusive assignments, but they are likely to be dimeric capsids with truncated genomes. The raw spectrum of the Figure 6 is available in the Supporting Information (Supporting Figure 3).

**Figure 6:**
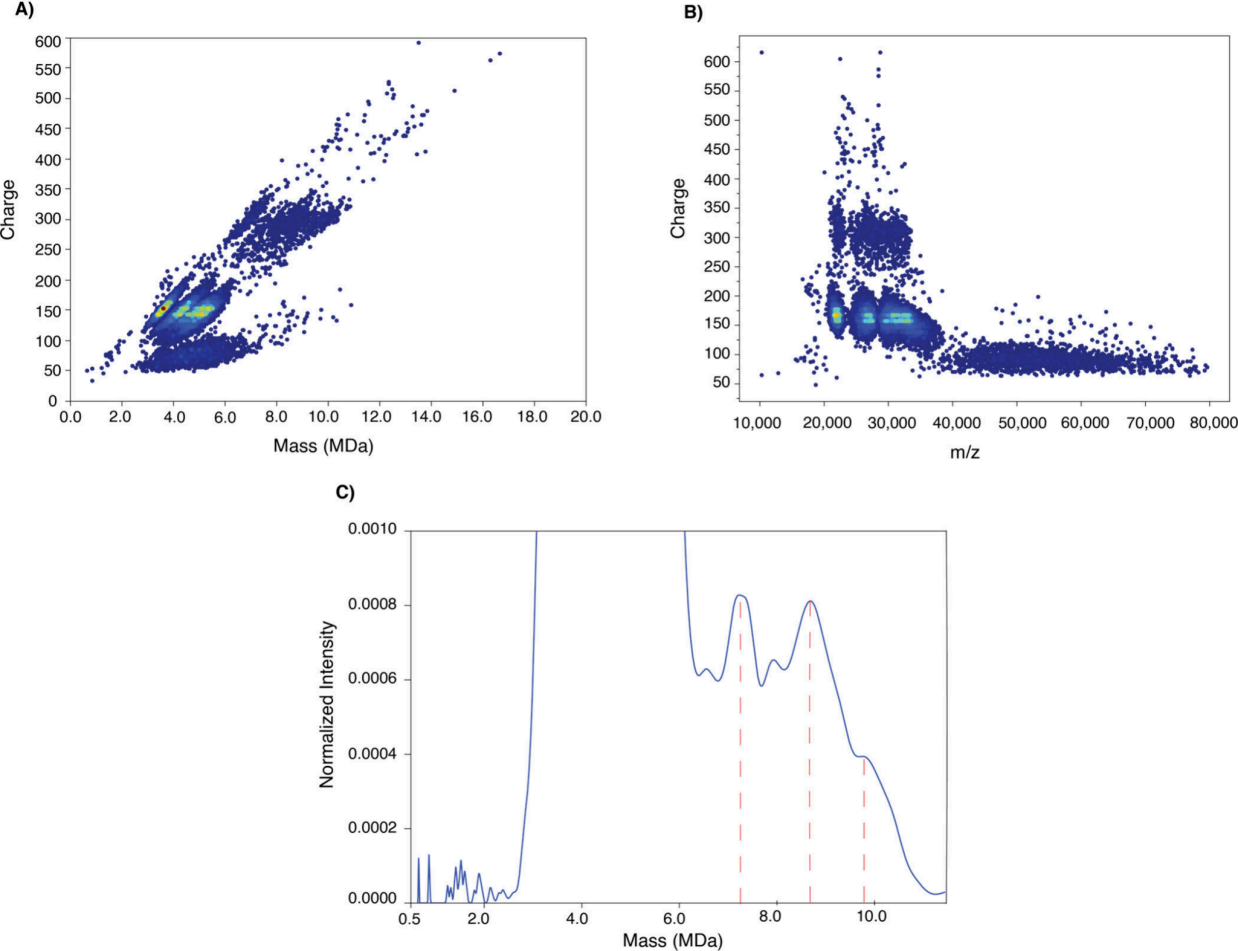
**CD-MS analysis of AAV8 capsid dimers (F2-nSEC).** (A) Charge versus mass distribution scatter plot of the F2-nSEC (AAV8 capsid dimers, *n* = 3). (B) Charge versus mass/charge distribution scatter plot of the F2-nSEC (AAV8 capsid dimers, *n* = 3). (C) CD-MS spectrum (zoomed) of the F2-nSEC (AAV8 capsid dimers, *n* = 3).

Finally, we evaluated the efficiency of the nSEC purification step. We compared the CD-MS data from the F3-nSEC sample with the whole sample mixture without nSEC purification. Both samples were analyzed at the same concentration (triplicate measurements). As illustrated in Figure 7 and Table 1, a higher percentage of empty capsids was found in the whole sample mixture, while the percentage of full capsids was found to be higher in the F3 sample. Since the filled particles were found to carry a small portion of empty particles, it is not a surprise that both the whole sample mixture and the F3 sample have higher percentages of empty particles. It is worth noting that this finding is consistent with the nSEC ratios. Importantly, the ratio (empty:full) of the F3 sample correlates well with the true 1:1 ratio, which reflects a more accurate measurement of the capsid content. Thus, our data confirms that nSEC and CD-MS are complementary tools for a more precise AAV quantification. The raw spectra of the Figure 7 is available in the Supporting Information (Supporting Figure 4).

**Figure 7:**
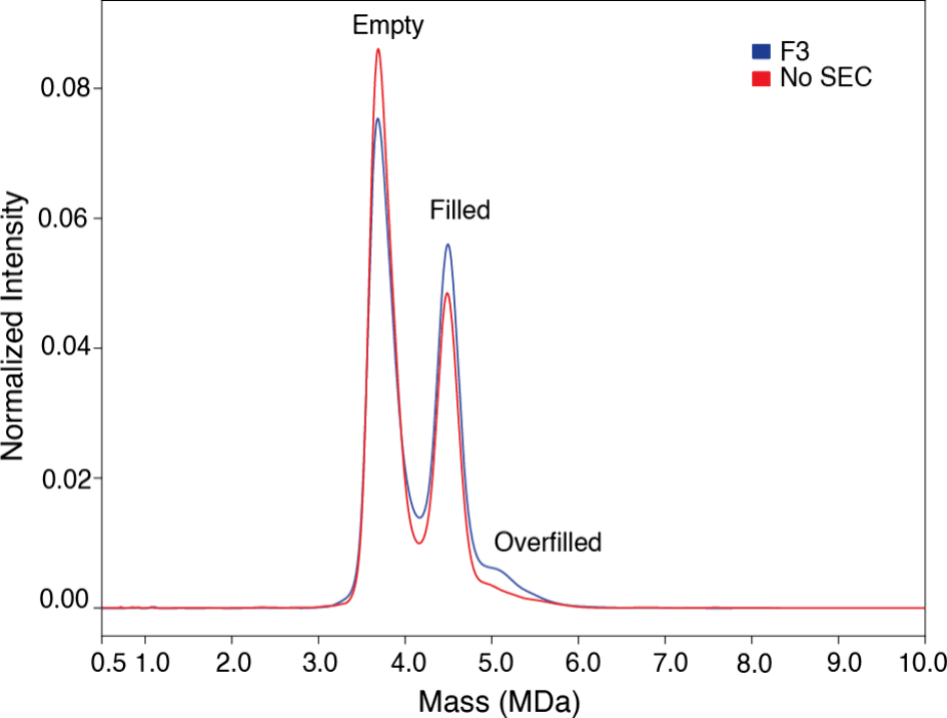
CD-MS analysis of the AAV8 mixture (empty:filled) with the sample F3 (blue) and the whole sample mixture (red) at the same concentration.

## Conclusions

Our findings clearly demonstrate that our strategy is well-suited to reliably and precisely assess the ssDNA content in AAV products by determining vector’s subpopulations (empty, filled, and with two truncated genomes). Along with nSEC, CD-MS provides detailed information about AAV8 capsid content and structure. Our nSEC strategy can separate the AAV8 capsid monomers from oligomers, higher-order aggregates, and low molecular weight fragments. MALS enabled the detection of low-abundant AAV8 oligomers and higher-order aggregates.

Importantly, the nSEC step ensured an accurate determination of the empty:filled capsid ratio. CE infusions provided remarkable spray stability and ensured the reproducibility, robustness, and sensitivity of the orbitrap-based CD-MS method. CD-MS confirmed the presence of oligomers and the mass of encapsidated genome without the need of pretreatment. It also distinguished empty from filled and overfilled capsids, including truncated genomes. This reflected the high degree of complementarity between these two strategies. Aggregation, oligomerization, and genome truncation in AAV formulations are problems of significant concern, affecting the safety and efficacy of AAV-based gene therapies.^38,48^ Analysis of the fractions containing AAV8 capsid monomers and oligomers by CD-MS revealed AAV8 capsid monomers and dimers with different conformations. The influence of these structures in the assembly of AAV capsids remains to be elucidated. Identification of empty and overfilled AAV capsids is of key importance for the development and production of AAV-based therapies. In addition to contributing to the total viral load without providing any therapeutic benefit, empty and overfilled capsids might overestimate the therapeutic potency of AAV-based therapies because the total particle count might not accurately reflect the number of functional AAV capsids. During clinical dosage, empty vectors add to the total viral load, and capsid-triggered immune responses might be worsened by increased viral loads. Regardless of their value, overfilled, filled, and empty capsids must be closely monitored to ensure the quality and efficacy of AAV-based therapeutics. This workflow enables quality and structural assessments of AAV products, and its implementation can guide AAV production for the safety and efficacy of AAV-based drugs.

## Supplementary Material



## Notes

Supplementary figures, including the raw spectrum of Figure 3 (F3-nSEC, capsid monomers); raw spectra of Figure 5A,B (empty and filled capsids without nSEC purification); the raw spectrum of Figure 6 (F2-nSEC, capsid oligomers); and raw spectra of F3-nSEC and the whole sample mixture at the same concentration (Figure 7) (PDF)
