# Supplementary material for: Characterizing the Content and Structure of AAV Capsids by Size Exclusion Chromatography and Orbitrap-Based Charge Detection-Mass Spectrometry
Source: J Am Soc Mass Spectrom. 2025 Jun 26;36(8):1659–68. doi: 10.1021/jasms.5c00074 (PMC12333375; doi:10.1021/jasms.5c00074)
Supplement: Supplementary file 1 [file js5c00074_si_001.pdf]

Supporting Information

Characterizing the content and structure of AAV capsids by size exclusion chromatography and orbitrap-based charge detection mass-spectrometry

Kanchan Pathak[1], Gustavo Perrotti[1], Stephen J. Rosa[2], Graham Robinett[2], Lance Kasper[2], Qiangwei Xia[3], Carlos R. Escalante[4], and Fabio P. Gomes[1]*

Kanchan Pathak[1], Gustavo Perrotti[1], Stephen J. Rosa[2], Graham Robinett[2], Lance Kasper[2], James Qiangwei Xia[3], Carlos R. Escalante[4], and Fabio P. Gomes[1]*

[1]Virginia Commonwealth University, Department of Chemistry, Richmond, VA 23284;
[2]Agilent Technologies, Santa Clara, CA 95051;
[3]CMP Scientific Corp, Brooklyn, NY 11226;
[4]Virginia Commonwealth University, School of Medicine, Department of Physiology and Biophysics, Richmond VA 23298.

Corresponding Author *(F.P.G.) Email: pereiragomf@vcu.edu

Table of Contents

Supplementary Figures

- **Supplemental Figure 1. Raw spectrum of the Figure 3 (F3-nSEC, capsid monomers).** The raw spectrum is averaged (10min).
- **Supplemental Figure 2. Raw spectra of the Figures 5A-B (empty and filled capsids without nSEC purification)**. The raw spectrum of the empty capsid is in black, and the raw spectrum of the filled capsid in in red. The raw spectra are averaged (10min).
- **Supplemental Figure 3.** Raw spectrum of the Figure 6 (F2-nSEC, capsid oligomers). The raw spectrum is averaged (30min).
- **Supplemental Figure 4. Raw spectra of the F3-nSEC and whole sample mixture at the same concentration (Figure 7).** The raw spectrum of the F3-nSEC is in black (top), and the raw spectrum of the whole sample mixture is in in red. The raw spectra are averaged (10min).

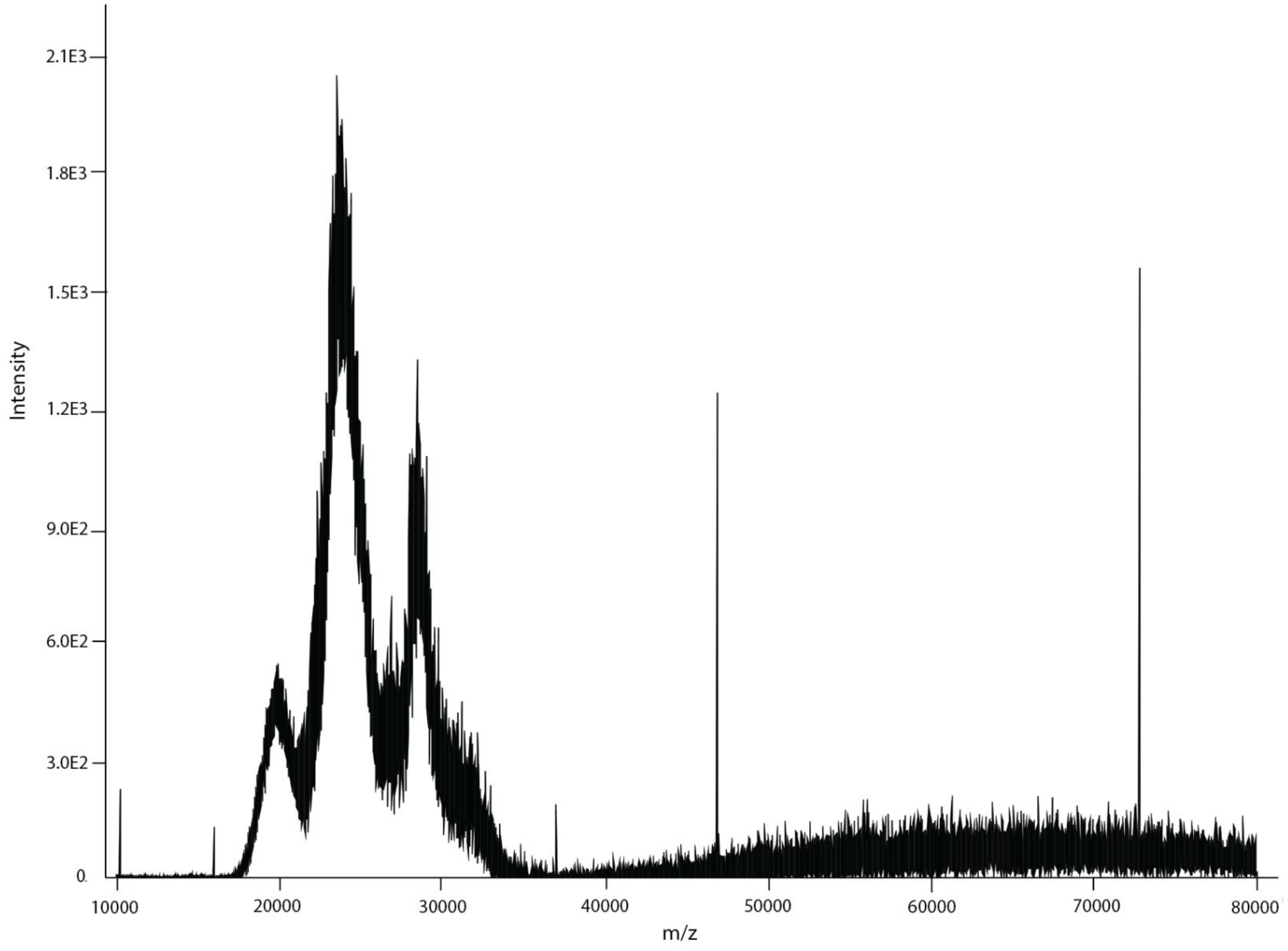

Supplemental Figure 1. Raw spectrum of the Figure 3 (F3-nSEC, capsid monomers). The raw spectrum is averaged (10min).

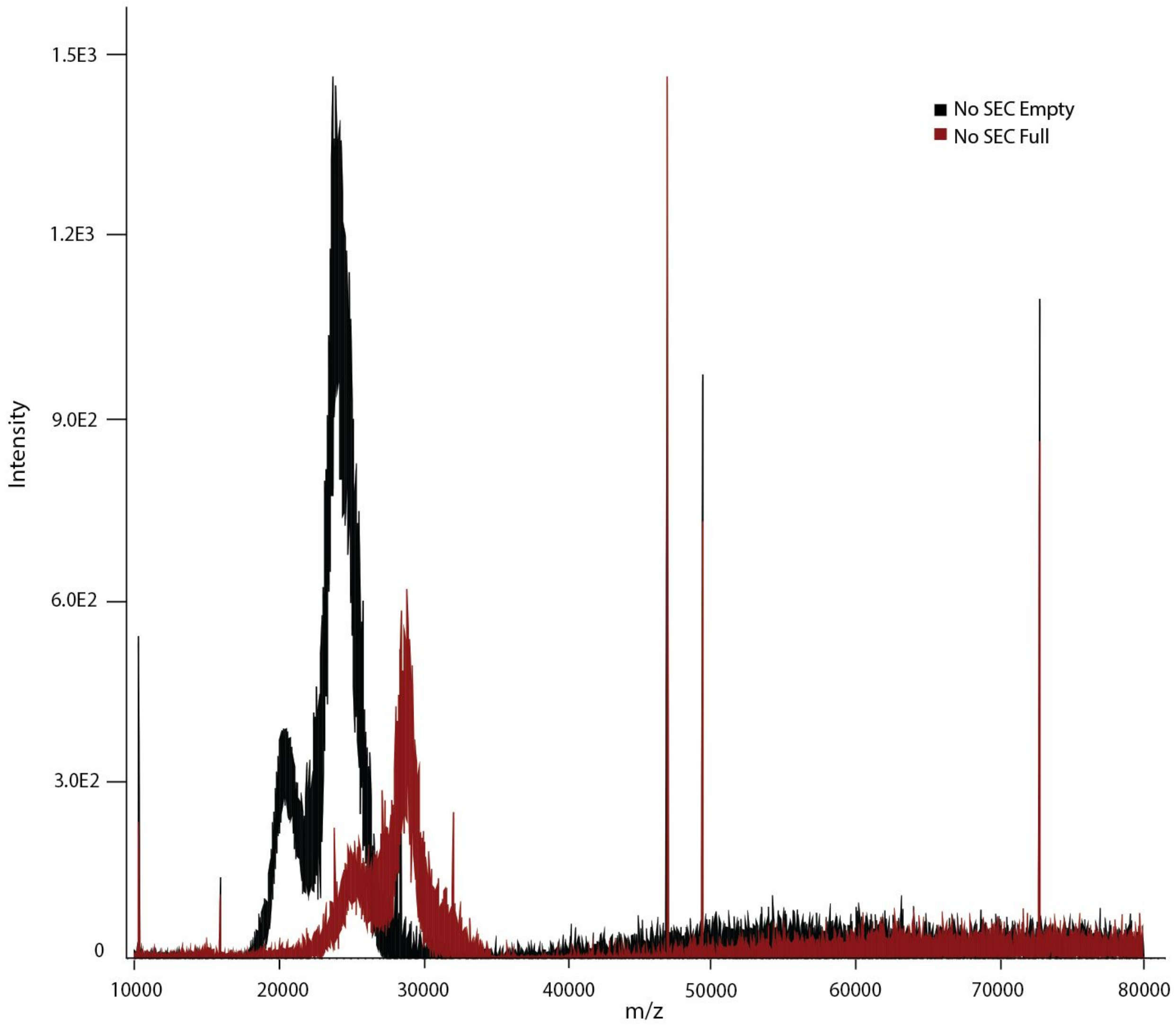

Supplemental Figure 2. Raw spectra of the Figures 5A-B (empty and filled capsids without nSEC purification). The raw spectrum of the empty capsid is in black, and the raw spectrum of the filled capsid is in red. The raw spectra are averaged (10min).

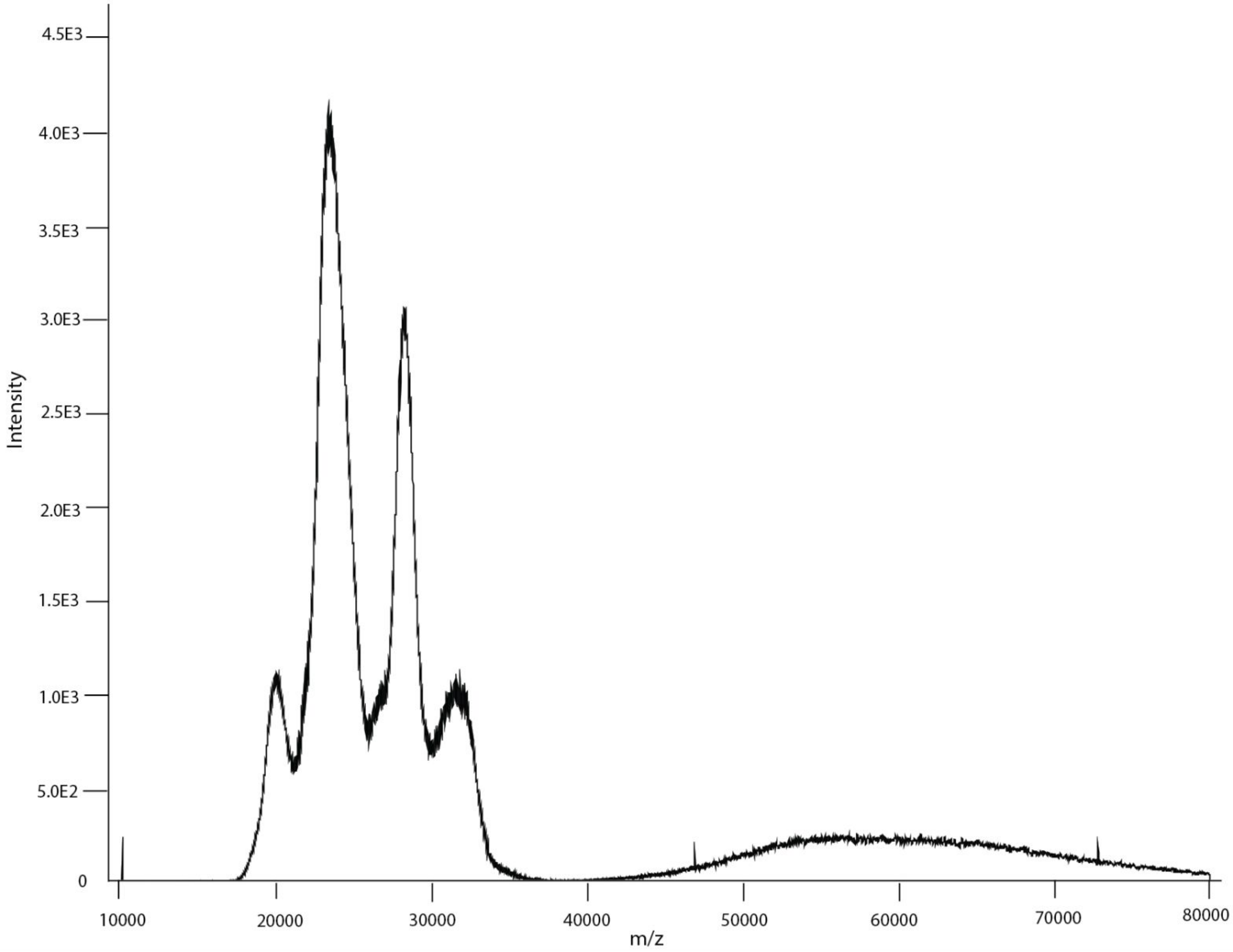

Supplemental Figure 3. Raw spectrum of the Figure 6 (F2-nSEC, capsid oligomers). The raw spectrum is averaged (30min).

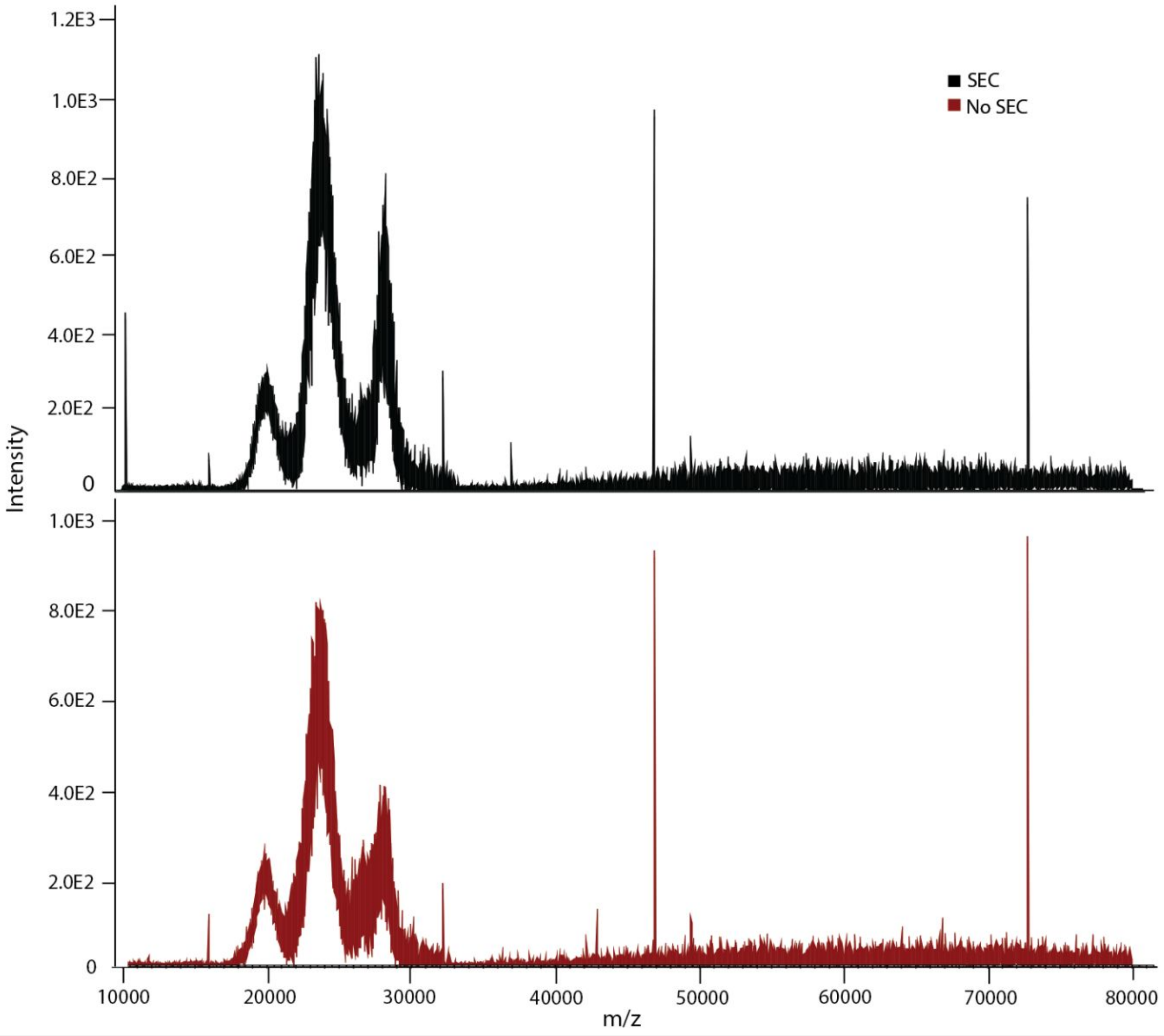

Supplemental Figure 4. Raw spectra of the F3-nSEC and whole sample mixture at the same concentration (Figure 7). The raw spectrum of the F3-nSEC is in black (top), and the raw spectrum of the whole sample mixture is in red. The raw spectra are averaged (10min).
